# Biogenesis and Mechanism of Action of Small Non-Coding RNAs: Insights from the Point of View of Structural Biology

**DOI:** 10.3390/ijms130810268

**Published:** 2012-08-17

**Authors:** Marina C. Costa, Ana Lúcia Leitão, Francisco J. Enguita

**Affiliations:** 1 Instituto de Medicina Molecular, Faculdade de Medicina, Universidade de Lisboa, Av. Prof. Egas Moniz, Lisboa 1649-028, Portugal; E-Mail: marinacosta@fm.ul.pt; 2 Departamento de Ciências e Tecnologia da Biomassa, Faculdade de Ciências e Tecnologia, Universidade Nova de Lisboa, Campus da Caparica, Caparica 2829-516, Portugal; E-Mail: aldl@fct.unl.pt

**Keywords:** non-coding RNA, structural biology, X-ray crystallography, microprocessor, miRNAs, Dicer, DGCR8, argonaute

## Abstract

Non-coding RNAs are dominant in the genomic output of the higher organisms being not simply occasional transcripts with idiosyncratic functions, but constituting an extensive regulatory network. Among all the species of non-coding RNAs, small non-coding RNAs (miRNAs, siRNAs and piRNAs) have been shown to be in the core of the regulatory machinery of all the genomic output in eukaryotic cells. Small non-coding RNAs are produced by several pathways containing specialized enzymes that process RNA transcripts. The mechanism of action of these molecules is also ensured by a group of effector proteins that are commonly engaged within high molecular weight protein-RNA complexes. In the last decade, the contribution of structural biology has been essential to the dissection of the molecular mechanisms involved in the biosynthesis and function of small non-coding RNAs.

## 1. Introduction

The central dogma of biology holds that genetic information normally flows from DNA to RNA and to proteins. As a consequence it has been generally assumed that genes code for proteins, and that proteins fulfill not only most structural and catalytic but also most regulatory functions in cells [1]. This is essentially true in prokaryotic organisms whose genomes are almost entirely composed of closely packed protein coding sequences. However, this is not the case in higher organisms in which proteomes and their coding sequences occupy only a tiny fraction of the genome. Around 97–98% of the transcriptional output of the human genome is non-protein coding RNA (ncRNA). This estimate is based upon the fact that intronic RNA constitutes 95% of primary protein coding transcripts (pre-mRNAs) and on a range of observations that suggest that there are a large number of ncRNA transcripts that do not contain substantial open reading frames and which may represent at least half of all the transcripts [2,3]. However, it is hard to escape the general conclusion that either the human genome is replete with useless transcription units, or that these RNAs are fulfilling some unexpected functions [4].

Many different ncRNAs with different functions in eukaryotic cell and developmental biology have already been described [5–7]. The fact that noncoding RNAs carry out the majority of the transcription of the genomes of humans and other complex organisms suggests that a second tier of genetic output and a network of parallel RNA-mediated interactions has evolved in these organisms, which may enable the integration and coordination of sophisticated suites of gene expression required for differentiation and development [8–10]. The expansion of the complement of ncRNAs in the higher organisms also suggests that the evolution of complexity may not have been simply dependent on an expanded repertoire of proteins and protein isoforms, but on a (much) larger set of genomic design instructions embedded in trans-acting RNAs (and cis-acting receiver sequences), which form the basis of a cascade of programmed response networks capable of implementing stored sequences of dynamical activities in response to internal and external stimuli. It is also likely that alteration in this control architecture is responsible for much of the phenotypic variation that is observed between individuals and species (such as vertebrates) that use a relatively common set of functional components, with the remainder of the variation (and the majority of catastrophic problems) due to variation in the components (the proteins) themselves [11,12]. Many ncRNAs are simply unprocessed primary transcripts but in other cases there are formed from the exons of spliced transcripts, which may also be alternatively spliced or polyadenylated. Some ncRNAs are derived from the further processing of exons such the miRNAs produced by some transcripts, and of introns of both protein coding and ncRNA genes as exemplified by snoRNAs.

**Figure 1:**
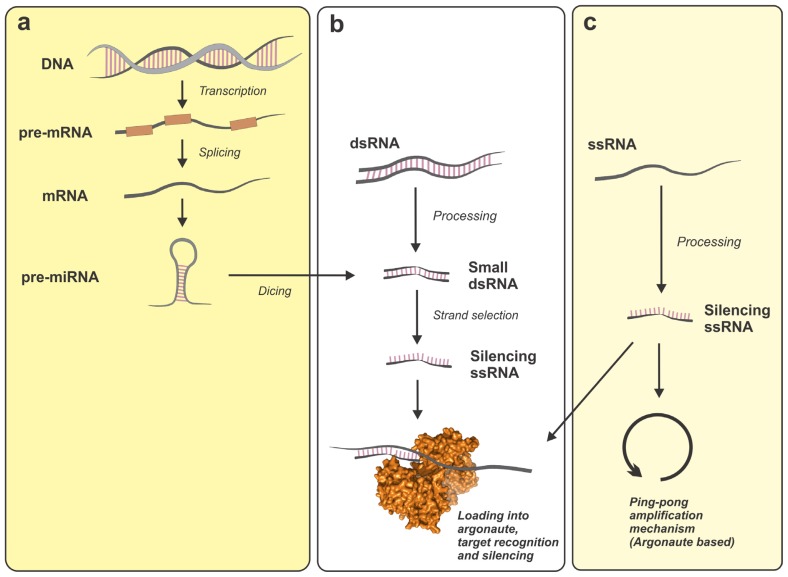
Pathways of small non-coding RNA generation in eukaryotic cells showing their main steps and relationships between groups. micro-RNA (miRNA) and small-interfering RNAs (siRNAs) are generated by nucleases (Drosha and Dicer) from dsRNA precursors, whereas PIWI-interacting RNAs (piRNAs) are produced from long single-stranded RNA precursors by an already not completely understood mechanism. (**a**) miRNA canonical pathway; (**b**) small-interfering RNA pathway; (**c**) piRNA pathway. dsRNA: double-stranded RNA; ssRNA: single-stranded RNA.

There are three major families of small non-coding RNAs (ncRNAs) in eukaryotic cells: micro-RNAs (miRNAs), piRNAs and small-interfering RNAs (siRNAs). These families are different in their origin, but they share specific steps in their biosynthetic pathways and regulatory mechanisms (Figure 1). In general, small ncRNAs are regulators of genomic output either at the transcriptional or at the post-transcriptional levels [13,14]. Regulatory control by small ncRNAs requires two main groups of proteins: processors and effectors. The processors are enzymes with nuclease activity able to excise small RNAs from specific RNA transcripts. On the other hand, the group of the effectors is constituted by a diverse cohort of RNA-binding proteins responsible for the stabilization, transport and regulatory activity of the small ncRNA over its cognate target [15].

Over the last decade, Structural Biology methods such as X-ray crystallography, NMR spectroscopy and more recently electron microscopy, have deeply contributed to the overall knowledge of the structure-function relationships among the components of the functional pathways of small ncRNAs [16–18]. In this review we will summarize these contributions, with special emphasis to the human key proteins involved in the biosynthesis and function of small ncRNAs.

## 2. Micro-RNAs (miRNAs)

Among non-coding RNAs, the best known family is constituted by miRNAs. These RNA molecules firstly discovered in the worm *Caenorhabditis elegans*, are short ncRNAs (21–23 nt) generated by a complex cellular pathway which starts with the transcription of specific genomic loci and flows from the nucleus to the cytoplasm with the help of specialized nucleases that cleave the RNA transcripts to produce a mature miRNA [19,20]. In the nucleus a tandem of proteins, Drosha and DGCR8, will recognize and excise small RNA hairpin loops formed during transcription in specific transcriptional units [21,22]. Drosha is a nuclease whereas DGCR8 recognizes the stem-ssRNA junction in pri-miRNAs. The excised loops called pre-miRNAs are exported to the cytoplasm through the nuclear pores with the help of Exportin-5 [23]. In the cytoplasm, the pre-miRNAs are recognized by a type III ribonuclease called Dicer that will cut the loop to generate a small dsRNA fragment of 19–23 pbs that will remain bound to the enzyme [24,25]. In this situation, the complex between Dicer and the small dsRNA will recruit several proteins of the argonaute family that will form the RNA induced silencing complex (RISC) that will select one of the chains of the dsRNA to generate a mature miRNA molecule [26,27]. Mature RISC complexes will be targeted to complementary sequences in mRNA transcrips, interfering with the translation process and consequently reducing the protein expression from mRNA transcripts [28–30]. In animal cells the complementarity of the miRNA and its target is not perfect, whereas in plants miRNAs are often 90–100% homologous to the target mRNAs. In this last case the RISC complex can induce mRNA targeted degradation, catalyzed by one of the argonautes, AGO2 protein [31,32]. An alternative pathway for the formation of miRNAs from intronic RNA transcripts has been also described. In this pathway, nuclear processing of RNA hairpins by Drosha/DGCR8 is skipped and the pre-miRNA are fully constituted by small introns that are excised exclusively by the splicing machinery. These miRNAs generated by skipping of the nuclear part of the native pathway are called Mirtrons [33,34].

Genes encoding miRNAs are frequently clustered together, and they are very abundant in the human genome having a preferential location within introns [35,36]. However it is already known that some of these miRNAs are localized in exons. Alternative splicing of these exons in a cellular or tissue specific manner could control the production of some miRNAs that will regulate different genes also in a specific way.

Regulatory activity exerted by miRNAs is a complex process in which a single mRNA transcript can be targeted by several miRNAs at the same time, and also a single miRNA can regulate hundreds of different mRNA targets [37–39].

## 3. piRNAs

Many organisms including mammals produce short-RNAs to regulate gene expression. piRNAs derive from long single-stranded RNAs transcribed from specific regions within the genome. In fact, piRNAs are generated from regions harboring transposons, and they were firstly described as a mechanism to protect the cells against the internal attacks of transposons [40]. They are preferentially expressed in germinal lines, however recent evidences showed that they could have expanded regulatory roles also in somatic cells [41–44]. piRNAs are able to interact with a specialized family of argonaute proteins called PIWI that will guide them to their targets and silence the transposon transcripts by their slicing activity [45,46].

piRNAs are slightly longer than miRNAs (24–31 nt in length), typically 3′-methylated and their biogenesis is still not well understood. The fact is that piRNAs are generated from longer RNAs transcribed from direct and reverse DNA strands from transposon regions, and sliced into small mature piRNAs by a mechanism that in *Drosophila* involves different proteins in somatic and germ lines. In somatic cells, the piRNA precursor is cleaved into mature piRNAs by a coordinated reaction involving the putative helicase Armitage and the putative nuclease Zucchini [44,47]. In germ cells, the biogenesis of piRNAs is dependent of two proteins, AUB and AGO3. Mature piRNAs captured by AGO3 will recognize their targets that will be cleaved by the AGO3 slicer activity and subsequently the generated fragments will constitute secondary piRNAs that amplify the signaling loop in a “ping-pong” mechanism [48–50]. This simple mechanism allows a rapid and efficient transposon silencing in germ cells even with a small amount of generated primary piRNA [51–53]. Some authors considered that the transposon rich-clusters in the genome together with the ping-pong amplification cycle constitutes a RNA-based immune system against RNA threads [54].

piRNA are considered as the precursor of an ancient mechanism of defense against genetic threads suffered by cells. In fact these small non-coding RNAs have been also found in primitive organisms such as cnidarians and sponges [55]. Despite their relatively well understood role in germ lines, the functions of piRNAs in somatic cells are still far from being understood, and more investigation is needed.

## 4. siRNAs

The small-interferring RNAs (siRNAs) are small dsRNAs generated by Dicer from longer precursors [56]. They were first described and further characterized in worms and plants as products of the catalytic action of RNA-dependent RNA polymerases (RdRPs) [57–59]. RdRPs are able to generate long dsRNAs that will be subsequently exported to the cytoplasm, processed by Dicer and recruited to the specific target by the cytoplasmic silencing complexes [60–62]. In plants, siRNAs are sometimes produced as a response to an external stress or as defense mechanism against genetic mobile elements or viruses [63,64].

However, in animals the apparent absence of RdRPs in their genomes prevented the search for endogenous siRNAs until the accidental discovery of LINE-1, a retro-transposon detected in human cell cultures able to produce a bidirectional RNA transcript using a double promoter system in the sense and antisense orientations [65,66]. In flies, next generation sequencing data of RNA pools obtained from AGO2 immunoprecipitation allowed also to identify a siRNA population clearly distinguisible from the miRNAs and piRNAs. Small interfering-RNAs from *Drosophila* are 21 nucleotides long, have modifications at the 3′ ends and are double-stranded [45,67]. In many cases, siRNAs are related with pseudogenes containing regions with tandem inverted repeats that allow the formation of long intramolecular dsRNA structures susceptible to be processed by Dicer [45].

Moreover, siRNAs have been also identified in mouse oocytes. As in flies, mouse siRNAs are 21 nucleotide long and Dicer-dependent products and in some ocasions their targets are within protein coding genes [40,60]. However, the regulatory functions of siRNAs are still not clear in the eukaryotic context. They probably have evolved from a more primitive defense system, and some authors consider that the transcriptional units producing siRNAs are still under evolutionary pressure [55,68]. Indeed, the key challenge will be to understand the real role of endogenous siRNA, more specifically those that will target protein coding genes and how they regulate mRNA expression.

## 5. Small Non-Coding RNA Processors

Typically, small ncRNAs are generated from bigger RNAs by the help of specific endonucleases. These endonucleases belong to the RNAse III family, and constitute hot-spots in the production of small ncRNAs. Small ncRNA processing enzymes are modular proteins, harboring domains for the binding and recognition of the RNA precursor and nuclease domains for its processing [69,70]. In plants and mammals, the small ncRNA production is compartmentalized by the eukaryotic cell structure in two different locations: nucleus and cytoplasm.

### 5.1. Drosha and the Nuclear Microprocessor

The first step in the biosynthesis of miRNAs in animal and insect cells is catalyzed by a tandem of proteins that form the microprocessor complex: Drosha, a type III RNAse and DGCR8, a dsRNA-binding protein responsible for the recognition of specific hairpin loops [20,71]. These two proteins represent the essential requirement for the initial processing of pri-miRNA transcripts [21]. However, in human cell extracts, Drosha has been described as the core of two types of microprocessor complexes [72,73]: a binary complex composed only of Drosha and DGCR8 with a consistent pri-miRNA processing activity, and a second larger complex containing Drosha, DGCR8 and several accessory proteins [72,73]. It is not clear whether this bigger complex is assembled, because some of the accessory factors include hnRNP proteins, the dead box helicases DDX5 and DDX17, and the p68 and p72 proteins [71,72,74]. In plants, this binary microprocessor complex is substituted by a nuclear Dicer-like protein, DCL1, a type III RNAse that generates precursor dsRNAs from longer transcripts [75].

The involvement of Drosha in the nuclear processing of pri-miRNAs to generate pre-miRNAs was discovered by Lee and coworkers in 2003 [76]. A year later, the partner of Drosha (Pasha) was discovered in *Drosophila* defining the so-called “microprocessor” [77]. RNA interference experiments of both elements of the microprocessor produced an accumulation of pri-miRNAs in the cell nucleus, suggesting their pivotal role in miRNA generation [77]. In humans, DGCR8 protein was determined to be the partner of Drosha and core component of the microprocessor [72]. Interestingly, deletions in the chromosome 22 that affect the gene encoding DGCR8 protein are the cause of the DiGeorge syndrome and have been well known since the early 80s [78,79]. The discovery of the relationships between the locus encoding DGCR8 protein and the miRNA processing suggested that one of the main causes of DiGeorge syndrome is an impairment in miRNA biogenesis [72].

**Figure 2:**
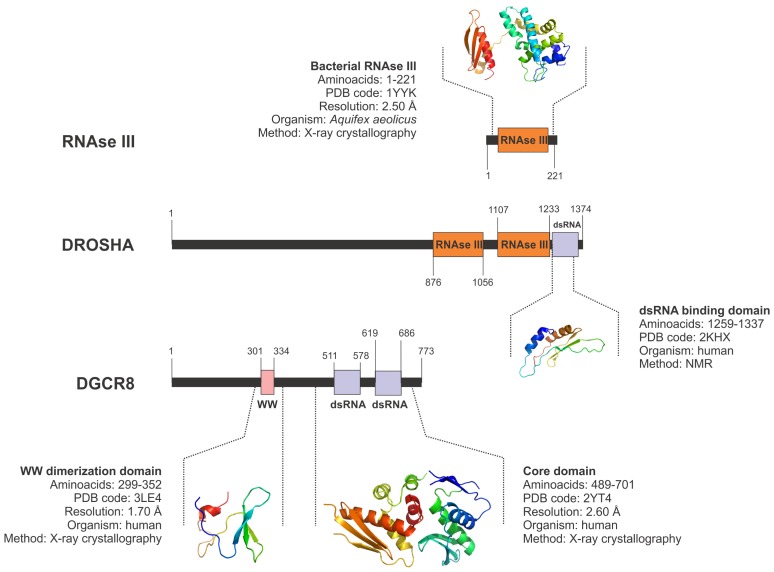
Domain map and available structural information of the human microprocessor components, Drosha and DiGeorge critical region 8 (DGCR8), and the bacterial RNAse III. Colored boxes along the sequence represent the functional domains present in each protein. The regions with already known three-dimensional structure are depicted, including information about the flanking aminoacids, PDB code for the atomic coordinates, resolution of the data used for refinement of the models, protein source and the experimental technique employed to determine the structure.

In humans, Drosha has 1374 aminoacid residues that can be clearly divided in two regions: a N-terminal segment from aminoacids 1 to 550, highly unstructured and flexible and probably involved in protein-protein interactions with other partners of the microprocessor complex; and the C-terminal segment from aminoacids 550 to 1374 that comprises two catalytic RNAse III domains and a terminal dsRNA binding domain. RNAse III domains in Drosha have been characterized on the base of their sequence homology with bacterial enzymes of the same family. The presence of a highly disordered N-terminal region has prevented the enzyme to be studied by X-ray crystallography (Figure S1). Until now, the only structural data from Drosha came from NMR experiments that determined the tridimensional arrangement of the C-terminal dsRNA binding domain (Figure 2) [80]. Because of the nature of Drosha, further structural studies to characterize the enzyme should probably employ a different approach combining methods such as NMR and electron microscopy to determine the structure of the enzyme in complex with other proteins.

On the other hand, DGCR8 is an RNA binding protein that in humans has 773 aminoacids. In this protein, the structure of two regions has been determined by X-ray crystallography: a WW-dimerization domain located close to the N-terminal region of the protein [81], and a dsRNA binding region comprising two different dsRNA binding domains and located in the C-terminal segment (Figure 2) [80,82]. The dsRNA binding domains showed a tridimensional structure composed of an alpha-helical core fused to a small beta-sheet segment that is arranged in tandem to ensure a wider coverage of the target RNA molecule [81,82].

Unexpectedly, DGCR8 has been characterized as a heme-binding protein, being this cofactor directly involved in the efficiency of pri-miRNA processing by the nuclear complex [83–85]. In fact, spectroscopic analysis of recombinant DGCR8 showed that it is the first known example of a heme-containing protein that harbors two axial cysteine ligands to complex a ferric iron [85]. The heme binding-motif is embedded as an independent region within the dimerization domain, suggesting also a contribution to the DGCR8 monomer interactions [81]. However, the exact role of the heme cofactor in the miRNA biosynthetic pathway remains elusive and further investigation is needed.

### 5.2. Cytoplasmic Processors: Dicer

The molecular mechanism of miRNAs and siRNAs is exerted mainly in the cytoplasm over mRNA transcripts. Dicer is an enzyme with a complex duty; first it has to capture pre-miRNA loops exported from the nuclear processing machinery by recognizing the end of dsRNA, cleave them to generate 21–23 nt dsRNAs, and second, it has to act as a meeting point for the recruitment of the proteins involved in the gene silencing (RNA silencing complexes) [86–88].

**Figure 3:**
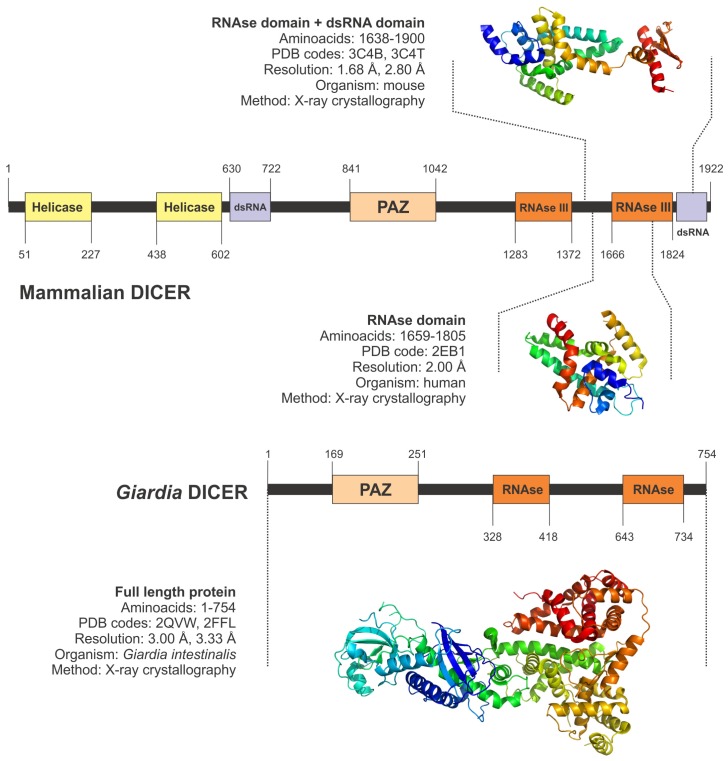
Domain map and available structural information of the mammalian and *Giardia* Dicers. Colored boxes represent the functional or structural domains present in each protein. The regions with already known tridimensional structure are depicted, including information about the limiting aminoacids, protein source, PDB code of the atomic coordinates, resolution and the experimental technique used to determine the structure.

Dicer-like proteins are well conserved in the entire eukaryotic world, suggesting a decisive role of these enzymes in the cell physiology. However, in complex eukaryotic cells such those belonging to mammals, Dicer proteins have evolved in a composite way comprising several duplicated domains that will perform all the protein functions (Figures S2 and S3). The most complete structural information available for a Dicer protein comes from the *Giardia intestinalis* protein. That is in fact a primitive Dicer, that has about half of the size of the human protein, and only contains two catalytic domains (RNAse domains) and a RNA binding domain (PAZ domain) (See Figure 3 for details) [89,90]. Data obtained from X-ray crystallography experiments in *Giardia*’s Dicer, allowed to determine that the protein acts as a molecular ruler, measuring the distance from the end of a precursor dsRNA and covering approximately 23–25 nucleotides, which is the distance between the PAZ domain and the catalytic RNAse region [89–91]. Dicer can process dsRNAs in a sequence-independent manner, allowing base pairing mismatches and different internal RNA structures. In fact this is a physiologically relevant feature of the enzyme, which allows virtually any dsRNA to enter the gene silencing pathways. The only exception to the general rule is the dsRNA lacking an open terminal region, which Dicer cannot process [91]. Interestingly, the analysis of the protein from *Giardia* revealed an important conformational flexibility driven by the presence of a flexible hinge in the interface regions between PAZ and RNAse domains. Authors have proposed a model of “induced folding” for the enzyme in response to the presence of a dsRNA substrate, in which the molecular hinge will adapt the PAZ and RNAse domains to embrace the RNA chain [89,91,92].

Human Dicer is a molecular machine with a much more complex structure than the *Giardia*’s protein (Figure 3). It conserves the catalytic core of PAZ and RNAse III domains, but also includes two dsRNA binding domains and a tandem of helicase domains in its N-terminal region (DExD domain). The helicase domains are also present in fly, worm, plants and yeast Dicers, but its function in the whole catalytic process remains unclear, being suggested to be involved in the discrimination of dsRNAs termini to promote an altered reaction mode [93]. Recent structural studies using a combination of electron microscopy with X-ray crystallography data allowed us to determine that the helicase domains in human Dicer are arranged in a clamp-like shape close to the RNAse III active site [70]. This architecture is also conserved in the protein from *Drosophila*, and is expected to appear also in other complex Dicers. The possible function of the helicase clamp is related with the recognition of pre-miRNA hairpin loops and long non-coding RNAs [70,94]. The absence of the helicase domain apparently does not affect the catalytic efficiency of human Dicer.

Recently, the X-ray structure of a catalytic fragment of mouse Dicer has been reported. Results showed the presence of a highly conserved lysine residue in the boundary of RNAse III domains that is involved in the dicing mechanism. In fact this catalytic lysine has been also described in other related RNAse III enzymes such as Drosha [95].

## 6. Efectors of the Small Non-Coding RNA Regulatory Networks: Proteins from the Argonaute Family

Argonaute proteins are widespread family of RNA-binding proteins described in organisms ranging from bacteria to humans, and always present in the core of RNA silencing complexes. Members of the argonaute family are able to bind small guide RNAs and to direct them to specific RNA transcripts for silencing or degradation of the messenger signal. In eukaryotic organisms, argonaute proteins are main effectors of the RNA-dependent regulatory machinery, being important players in the RNA interference, miRNA regulation, and piRNA function. Some eukaryotic argonautes harbor nuclease activity, being able to directly slice or degrade specific RNA molecules. Moreover their roles in prokaryotic organisms are still far from being clarified; however, it is assumed that they could play an important role in the defense systems against external genetic threads, namely bacteriophages and more recently mobile genetic elements [96].

Structurally, all the proteins belonging to the argonaute family consist of a highly variable N-terminal domain and three conserved domains: the PAZ domain, the MID or intermediate domain, and the PIWI domain. PAZ and MID domains are involved in the proper recognition and interactions with the small guiding RNA. PAZ domain interacts with the 3′ end of the small RNA and MID domain is responsible for the recognition and interaction with the phosphate group at the 5′ end of the small non-coding RNAs. Meanwhile the PIWI domain will guide the RNA-AGO complex to the target RNA.

Argonaute family of proteins is divided in three different phylogenetic subfamilies: the AGO subfamily, named after the discovery of Argonaute 1 protein in *Arabidopsis thaliana*; the PIWI subfamily, firstly described in *D. melanogaster* (P-element induced wimpy testis); and the WAGO subfamily (Worm-specific AGO proteins), only present in *C. elegans*.

### 6.1. AGO Sub-Family

AGO proteins are present in different extend from budding yeasts to mammals. They constitute the core of the RNA induced silencing complexes in the cytoplasm (RISC complex) and in the nucleus (RITS complex) [97,98]. Moreover, AGO proteins could be consider as a molecular bridge since their function requires a simultaneous interaction with RNA and proteins. In humans and other vertebrates there are four AGO paralogs designated as AGO1-4, harboring the characteristic domain structure of the argonaute family members (Figure S4). Among them, AGO2 is unique since it is the only member of the family able to catalyze selective RNA slicing *in vivo* as the functional core of the cytoplasmic RISC complex [99]. This slicing activity is only exerted when the complementarity between the small guiding RNA to its cognate target is almost complete. On the other hand, AGO1 is an essential component of the nuclear RITS complex in yeasts, regulating the chromatin structure in response to the presence of small non-coding RNAs complementary to nascent mRNA transcripts [100,101]. Functional analysis of human AGOs using epitope-tagging techniques has shown that the population of small non-coding RNAs that bind to AGO1 and AGO2 are different, suggesting a diversity in their target specificities [102]. The other members of the family, AGO3 and AGO4 are not well characterized in terms of function and specificity of action. Recently, AGO3 has been pointed out as a backup system for channeling miRNA action in the absence of AGO1 and AGO2 [103]. Taking into consideration all these facts and aside from these specialized functions, all mammalian Argonautes appear to cooperate in the small non-coding RNA pathways in a largely redundant and overlapping way.

#### 6.1.1. AGO2

Classical structural studies of full-length AGO proteins were initially performed on the prokaryotic family members because of their favorable behavior for overexpression and purification in bacterial platforms [104–107]. However, a few preliminary studies using isolated protein domains also showed the high structural homology among the building blocks of the family [108]. Recently, the full-length crystal structure of human AGO2 has been determined at 2.3 Å resolution in complex with RNA [109].

**Figure 4:**
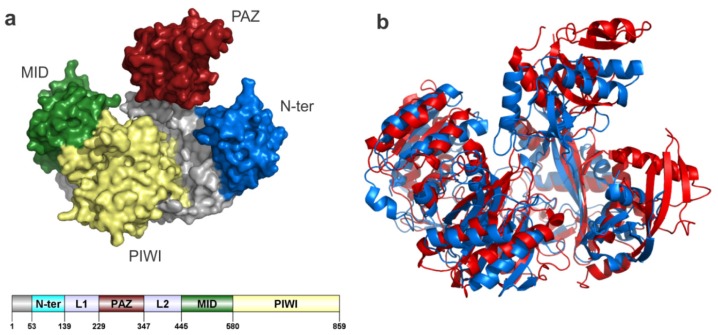
Structural elements of human AGO2 and comparison with prokaryotic members of the family. All the protein structure figures and diagrams were prepared with Pymol. (**a**) Molecular surface of human AGO2 with the four structural domains depicted in different colors, and relative position of the domains within the primary structure. (**b**) Three dimensional superposition of human AGO2 (red) and *P. furiosus* argonaute (blue) performed by the SuperPose server [115] and showing the relative position of secondary structure elements in both proteins. Sequence identity of both Argonautes is 17.0%. Global Root Mean Square Deviation (RMSD) of the three-dimensional superposition for a total of 657 alpha-carbons was 10.78.

Human AGO2 structure has the typical four domain arrangement also observed in other argonautes (Figure 4). N-terminal, MID and PIWI domains are organized to form a cavity that is partially covered by the PAZ domain lid. In comparison with its prokaryotic relatives, human AGO2 showed major architectural differences. Additional secondary structure elements present in the N-terminal, PIWI and PAZ domains will likely play a role in the recognition and binding of AGO-associated protein factors (Figure 4). However, structural alignment of human AGO2 (PDB code: 4EI1) and the argonaute protein from Pyrococcus furiosus (PDB code: 1U04) showed a common structural core that is evolutionarily connected also with the PIWI protein family [110].

Data obtained from the structural studies of bacterial argonautes mainly by Patel and coworkers, showed that argonaute proteins are carriers of small RNAs irrespective to the sequence, which is reflected in the absence of specific contacts between RNA bases and the protein chain [105–107,111]. Crystal structure of argonaute and the respective protein RNA complexes were determined at different resolution levels, taking advantage of the previous characterization of point mutants lacking RNA slicer activity [112,113]. Despite the natural preference of bacterial argonautes for DNA as guide strand instead of RNA, the determination of the structure of these complexes has increased the overall knowledge of its catalytic cycle. Indeed data from x-ray crystallography studies showed that bacterial argonautes embraces a two-state based mechanism for guide strand selection, target recognition and slicing.

The junction between the MID and PIWI domains is responsible for the formation of a binding pocket for the guide strand, that it is highly stabilized by interactions in the 5′ end of the nucleic acid chain. Moreover, the orientation of the guide strand allows its tethering of the 3′ end within the PAZ domain. Structural studies performed in mammalian PAZ domains isolated from full-length protein have confirmed the presence of a similar mechanism for attaching of the small RNA strand to the AGO2 pocket [108,114]. Interestingly, the recently solved structure of the human AGO2 showed the presence of electronic density across the MID-PIWI interface that the authors modeled as small cellular RNAs that remained bound to the protein along the purification and crystallization processes [109]. The modeled RNA is bound in a similar conformation that the DNA guiding strand found in the bacterial argonautes [109].

**Figure 5:**
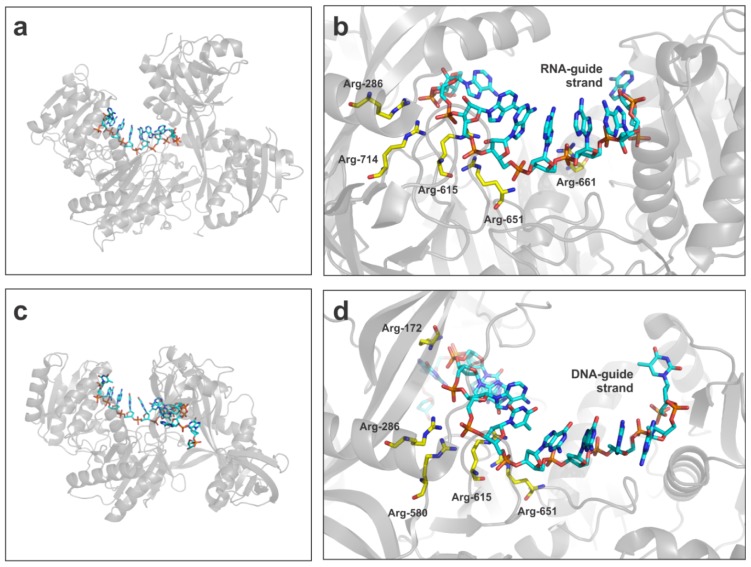
Mechanism of guide-strand capture by human AGO2 and bacterial argonaute from *P. furiosus* revealed by X-ray crystallography studies. (**a**) Overal folding of human AGO2 structure in complex with a RNA guide strand (PDB code: 4EI1). (**b**) Structure of the binding pocket of the RNA-guide strand in human AGO2 showing the arginine residues involved in the interaction with the phosphodiester backbone. (**c**) Overall folding of argonaute from *P. furiosus* in complex with a DNA guide strand (PDB code: 3DLH). (**d**) Structure of the binding pocket of the DNA-guide strand in *P. furiosus* argonaute with the arginine residues interacting with the negatively charged groups in the DNA skeleton. Arg-286, -615 and -651 are conserved between both structures.

The spatial orientation of the guide strand within the argonaute pocket will favor the molecular interactions with its cognate target. The nucleic acid guiding strand is oriented exposing the edges of the nucleotides from the seed sequence to the outer part of the protein, ready to capture a target. This orientation is accomplished by direct polar interactions between the phosphodiester skeleton and positive residues in the argonaute pocket. The seed sequence of the guide strand is captured in the core of the protein, meanwhile the 5′ end of the guide nucleic acid is in a flexible conformation, showing the absolute importance of the seed sequence in the global target recognition process [112,116,117] (Figure 5).

Comparison of key RNA interacting residues in human AGO2 versus bacterial argonaute showed a clear conserved pattern comprising Arg-286, -615 and -651 suggesting a similar mechanism of binding to RNA in both the members of the family (Figure 5). In human AGO2 the slicer activity is ensured by the presence of three catalytic residues, Asp-597, Asp-699 and His-807 [99,104]. Interestingly, the majority of argonaute proteins are catalytically inactive under physiological conditions, in despite of the relative conservation of the three active residues [99,102,118]. The underlying mechanistic explanations for this fact are still in the matter of the speculation, but probably there are additional external factors that would contribute to the slicer activity of AGO2 like post-translational modifications or helper proteins [103].

#### 6.1.2. Other Members of the AGO Sub-Family

In humans and other mammals, the argonaute sub-family comprises also three additional members named AGO1, AGO3 and AGO4. Their probable redundant functions in the cell are in contradiction with their RNA-binding specificity. In fact, RIP-seq studies with tagged human argonautes showed clear differences in the cohort of small non-coding RNAs that are bound to them [102]. Human AGO2 and AGO3 appeared to be specific for miRNA binding and targeting, whereas AGO1 is more specific for siRNAs [102,119,120]. On the other hand, the function of AGO4 has been mainly characterized in plants. In fact, in *Arabidopsis thaliana* AGO4 is responsible for the guiding of several DNA methyltransferases to chromatin [62,121,122]. Moreover, AGO4 in plants is also related with the silencing of repetitive genome elements by a mechanism involving also a long intergenic non-coding RNA (lincRNA) [123,124]. In mammals, a proposed cooperative model of argonautes action based on high-throughput sequencing data is currently arising [125].

As we previously pointed out, AGO1, AGO3 and AGO4 lack slicer activity over mRNA targets, however two of the catalytic residues Asp-597 and Asp-699 found in the nuclease active AGO2 are conserved in all the family members. The last residue of the catalytic center, His-807, is only conserved in AGO2 and AGO3, being substituted by an arginine in AGO1 and AGO4.

In order to determine some structural features of all AGO family members, we have performed a homology modeling to determine the predicted three dimensional structure of the human AGO family members using AGO2 atomic coordinates as a reference. Homology modeling is a well-established and reliable protocol for structure determination when the sequence homology is higher than 40%. Some recently developed methods such as Phyre algorithm take also into account secondary structure information in order to increase the probability to get a reliable tridimensional model [126,127]. Phyre was able to build tridimensional models of human AGO1, AGO3 and AGO4 with a 99.9% confidence over the 92% of the aminoacid sequence using AGO2 coordinates as a template (PDB code: 4EI3). Atomic coordinates of Phyre models for AGO1, AGO3 and AGO4 are available as supplementary files.

**Figure 6:**
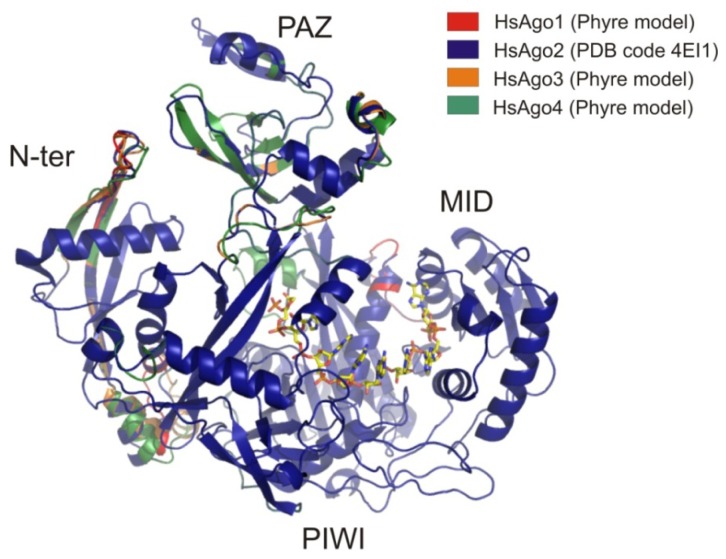
Structural superposition of the human AGO family members performed by the SuperPose server [115]. Homology models for AGO1, AGO3 and AGO4 were built with Phyre software [127] using coordinates of the human AGO2 as a template (PDB code: 4EI1). Structural domains are depicted in the figure. Calculated RMSD of the global structural alignment was 0.818 for all the alpha-carbons and 0.820 for the backbone aminoacid chains.

Aligned models generated by Phyre are represented in Figure 6. Observed structural differences among the human AGOs are mainly present in the N-terminal and PAZ domains. MID and PIWI domains are structurally very similar in all the members of the family. Additional secondary structure elements are clearly observable in N-terminal and PAZ domains in AGO4 and AGO3 proteins in comparison with the reference model (Figure 6). Since the RNA binding pocket is homogeneously conserved in all AGOs, we can hypothesize that the structural differences observed in PAZ and N-terminal domains could be related with their different partner specificity [71,128]. However, more detailed studies are required to understand the physiological role of all the AGO proteins and their target specificity.

## 7. Helper Proteins and Additional Members of the Non-Coding RNA Effector Complexes

### 7.1. TRBP

TAR RNA binding protein 2 (TRBP2) was discovered and characterized as a trans-activation responsive protein against HIV infection in humans [129,130]. In 2005 the group of Shiekhattar discovered the interaction of TRBP with Dicer and its ability to recruit AGO2 to form a RISC loading complex (RLC) [131]. The RLC is responsible for the selection of one of the strands of the small ncRNA to generate a mature RISC complex [26]. TRBP is a 366 aminoacid protein comprising three dsRNA binding domains in tandem. Its function appeared to be related with the recruitment of argonaute proteins to the mature RISC in a RNA-dependent manner [116,132,133]. In other organisms RLC contains R2D2, an ortholog of TRBP2 [134,135]. R2D2 protein senses the relative stability of dsRNA strands, selecting one strand depending on the hybridization energy and 3′-end of the strand [133]. Specifically, the strand with the less stable 5′ end at the siRNA duplex will be selected for loading the RISC complex and the other strand discarded being considered as a passenger strand.

Structural information of the isolated TRBP2 protein is limited to one of its dsRNA binding domains, determined by X-ray crystallography (PDB code: 3ADL) [136]. The structural features of the second dsRNA binding domain of the human TRBP2 are also observed in other similar domains and include a small strand segment flanked by two alpha helices [116,136]. In despite of the well-structured dsRNA binding domains, the connecting segments between domains are extremely flexible and have prevented the crystallization of the full length protein. However, more recent studies have characterized the RLC by cryo-electron microscopy; Lau and coworkers determined the structure of a TRBP2-Dicer complex at 20 Å resolution [137]. Docking of the X-ray structures of *Giardia*’s Dicer onto the obtained density, allowed the authors to exactly locate the position of the enzyme within the L-shaped complex [137]. More recently the group of Nogales, has characterized the whole RLC by electron microscopy showing that the complex is established on the basis of core Dicer interactions. In the RLC, Dicer interacts with TRBP2 by its N-terminal region and with AGO2 protein with the C-terminal catalytic domain [116].

### 7.2. GW182

In vertebrates, miRNA regulatory functions are exerted by the loaded RISC complex that it is recruited to its cognate mRNA targets. The partial complementarity of the miRNAs with their targets induces a translational repression of the mRNA and consequently reduced levels of translated protein. In *Drosophila*, the GW182 protein is recruited to the miRNA regulatory complex by its affinity to argonaute proteins. In Drosophila, GW182 protein has been characterized as a partner of the argonautes engaged in the RISC complex, able to interact with AGO proteins by a domain containing GW/WG repeats [138]. These molecular interactions are needed for a productive translational repression via degradation of the poly-A tail or simultaneous recruitment of the involved mRNA transcripts to P-bodies [136,137].

In humans and other vertebrates, there are at least three GW182 paralogs named TNRC6A, TNRC6B and TNRC6C with apparently redundant roles [139]. Proteins from the TNRC6 family showed a complex primary structure with several putative argonaute-interaction domains present along the aminoacid sequence. They also harbor a RNA-binding domain close to the C-terminal end of the protein and two glutamine-rich and glycine-rich regions in the N-terminal segment [118,139,140].

TNRC6B contains three binding sites for AGO2, within the amino-terminal glycine tryptophan (GW/WG)-repeated region that is characteristic of the GW182 family proteins. Multiple argonaute proteins are expected to be recruited to the RISC complex via interaction with GW182 protein [118,138]. Interestingly, X-ray crystallography experiments have demonstrated the direct interaction between TNRC6C and the cytoplasmic poly-adenine binding protein (PABPC1). This interaction is postulated to be directly involved in the deadenylation process and subsequent translational repression of mRNA transcripts induced by miRNAs [141,142]. However, the TNRC6C-PABPC1 does not have an observable deadenylation activity, it could be involved in the further recruitment of other specific enzymes that will catalyze the poly-A tail shortening and the transport of the selected mRNA to the P-bodies for degradation [143–145]. These interactions which are described as being critical for mRNA silencing by miRNAs are also conserved in *Drosophila* [144,145].

Structural information about GW182 family proteins will be limited by the intrinsic disorder propensity of all the members of the group. In fact, more than 60% of the aminoacids in TNRC6 group are predicted to be in disordered and present flexible regions. Disordered segments are concentrated in the first 500 residues of the protein (Figure S5), which is in agreement with the enrichment of argonaute-binding motifs in this region that will facilitate transient interactions with these proteins and different ways to build silence complexes [139,140].

### 7.3. PIWI Proteins

PIWI proteins were described because of their intrinsic binding affinity for piRNAs. The PIWI family of proteins is a sub-family of the argonaute group. In flies, the PIWI family is composed of Piwi, Aubergine (AUB) and AGO3 proteins; in mice MILI, MIWI and MIWI2; and in humans of HILI, HIWI1, HIWI2 and HIWI3 [146,147]. PIWI family is required for efficient transposon silencing in germinal cell lines, however they are also produced in somatic cells [67,148]. All the family members are similar in sequence to the AGO group, harboring the four characteristic argonaute domains N-terminal, PAZ, MID and PIWI.

The role of individual PIWI proteins has been extensively studied in *Drosophila*. In this model organism it is well documented that every PIWI protein has a different binding specificity for sense or antisense RNAs derived from transposon regions in the genome; PIWI and AUB have affinity for antisense transcripts, whereas AGO3 is mainly bound to sense RNAs [149]. Because PIWI proteins have slicer activity, any of them can initiate the “ping-pong” amplification cycle for transposon silencing already described in previous sections.

Structural information available about PIWI proteins is still limited in humans to the X-ray crystallographic structure of the PAZ domain of MIWI and HIWI proteins in complex with a single stranded nucleic acid (PDB code: 2XFM). PAZ domains from PIWI proteins are extremely similar to those observed in AGO proteins and also in Dicer [150].

Interestingly, recent observations described the presence of post-translational modifications in PIWI proteins. Arginine methylation has been characterized as a modulation mechanism to produce specific signatures of biological processes. In fact, several arginines present in the C-terminal domain of PIWI proteins are symmetrically dimethylated (sDMA) [148,149]. This arginine dimethylation could regulate the function of several proteins, including transcription factors and proteins belonging to the splicing machinery. In PIWI proteins, methylation of terminal arginines is catalyzed by PRMT5 methyltransferase. Methylated arginines are recognized by the Tudor family of proteins, classically linked to the gametogenesis process even before the discovery of PIWI proteins and piRNAs [151,152]. Tudor domains are protein modules that usually are mediating protein-protein interactions, potentially by binding to methylated aminoacids.

Recent published X-ray crystallography data, determined the molecular basis for the specificity of Tudor domain binding to methylated arginines in the C-terminal region of human MIWI protein [153]. Liu and coworkers determine that the specific recognition of dimethylated arginines by the Tudor domain is ensured by ionic interactions of a negatively charged groove in Tudor domain that recognize the positive charged arginine patch [152,153]. Additional data from X-ray crystallography experiments allowed also characterizing complexes between several additional proteins from the Tudor family. Chen and coworkers described the structure of the Tdrkh Tudor domain, and inferred its interaction mechanism with methylated arginines by mutagenesis analysis [154].

## 8. Other Helper Proteins: Exportin 5

Pre-miRNAs are actively exported from the nucleus to the cytoplasm to originate mature miRNAs. This transport is ensured by the Exp5-RanDTP system, firstly characterized by using *Xenopus* oocytes [155]. Exportin 5 is a ds-RNA binding protein that can translocate these molecules from the nucleus to the cytoplasm. This system is also capable of transporting other dsRNAs such as short-hairpin RNAs (shRNAs) [13,155,156]. RanGTPase-dependent export mediators (exportins) constitute the largest class of these carriers and are functionally highly versatile. As other exportins, Exp5 load its cognate pre-miRNA substrates in response to RanGTP binding in the nucleus and traverse the nuclear pores as ternary RanGTP-exportin-cargo complexes to the cytoplasm, where GTP hydrolysis leads to export complex disassembly [23,157,158].

**Figure 7:**
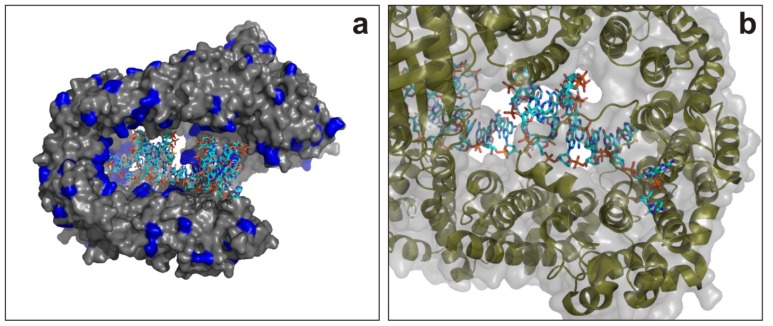
Crystal structure of Exportin 5 complex with a pre-miRNA. (**a**) Overview of the dsRNA-Exp5 complex with the positive charged residues (lysines and arginines) colored in blue (PDB code: 3A6P). (**b**) Detailed view of the protein-RNA interface with the 2-nucleotide 3′-overhang located in a narrow pocket of the protein.

The crystal structure of Exportin 5 in complex with a shRNA was determined by Okada *et al.* in 2009 [159]. The ternary Exp5-RanGTP-pre-miRNA studied by X-ray crystallography indicates that the interaction between pre-miRNA and exportin 5 is mainly driven through ionic contacts (Figure 7). A narrow pocket within the protein recognizes the two nucleotide 3′-overhang in the pre-miRNA allowing a specific interaction with the protein (Figure 7). Indeed, pre-miRNA blocking by Exp-5 in both its terminal nucleotides ensures enhanced protection for degradation against nucleases during transport.

## 9. Conclusions

Structural Biology has contributed to the global understanding of the regulatory mechanisms and biogenesis routes of small non-coding RNAs. Experimental data showed a group of proteins involved in the biogenesis and function of small ncRNAs, that are mainly modular. However the diversity of domains found in this family of proteins is limited, including only a small number of typologies: dsRNA binding, RNAse and helicase domains. These domains are core components of two different groups of enzymes: processors and effectors. Processor enzymes, with the exception of Drosha, are mainly well structured polypeptides. The group of the effectors is more diverse, and includes globular proteins but also polypeptides with long disordered regions that are involved in protein-protein interactions and consequently in complex stability.

Because of the inherent nature of the protein complexes involved in the regulatory functions of small non-coding RNAs, future studies will need to combine methodologies for the determination of tridimensional structures. Recent approaches joined together electron microscopy with X-ray crystallography and have been successfully applied to the characterization of RISC complexes. Further studies are needed to understand nuclear processing complexes and also to determine the role of protein-protein and protein-RNA transient interactions in the global landscape of non-coding RNA regulatory mechanisms.
